# Tuberculosis Infection Screening in 5468 Italian Healthcare Students: Investigation of a Borderline Zone Value for the QFT-Test

**DOI:** 10.3390/ijerph17186773

**Published:** 2020-09-17

**Authors:** Anna Rita Corvino, Maria Grazia Lourdes Monaco, Elpidio Maria Garzillo, Elena Grimaldi, Giovanna Donnarumma, Nadia Miraglia, Gabriella Di Giuseppe, Monica Lamberti

**Affiliations:** 1 Experimental Medicine Department, University of Campania “Luigi Vanvitelli”, 80138 Naples, Italy; annarita.corvino@unicampania.it (A.R.C.); elena.grimaldi@unicampania.it (E.G.); giovanna.donnarumma@unicampania.it (G.D.); nadia.miraglia@unicampania.it (N.M.); gabriella.digiuseppe@unicampania.it (G.D.G.); monica.lamberti@unicampania.it (M.L.); 2 Occupational Medicine Unit, University Hospital of Verona, 37134 Verona, Italy; mariagrazialourdes.monaco@aovr.veneto.it; 3 Department of Prevention, Abruzzo Local Health Authority, 67100 L’Aquila, Italy

**Keywords:** *Mycobacterium tuberculosis* infection, healthcare workers, QuantiFERON serial testing, LTBI

## Abstract

Healthcare workers are at an increased risk of contracting *Mycobacterium tuberculosis* infection. Tuberculin skin test (TST) and interferon gamma release assay (IGRA) represent the available tests most used for the diagnosis of latent tuberculosis infection (LTBI). Different borderline zones have been proposed for defining conversions and reversions to improve the interpretation of the IGRA test results as part of serial testing. From 2012 to 2017, 5468 health students of an Italian University Hospital were screened for tuberculosis infection through the execution of the TST and, in case of positivity, of the QuantiFERON-TB^®^ Gold In-Tube assay (QFT–GIT). The QFT–GIT is considered “borderline” with values from 0.35 to 0.99 IU/mL. Among the students who performed the QFT–GIT assay, 27 subjects presented a range of values defined as borderline. The QFT–GIT was repeated after 90 days on 19 subjects with borderline values and showed a negativization of the values in 14 students and a positive conversion in three cases, while for two students, a borderline value was also found for the second test, with a 74% regression of the borderline cases. The introduction of QuantiFERON borderline values is a useful assessment tool to bring out LTBI case candidates for chemoprophylaxis.

## 1. Introduction

*Mycobacterium tuberculosis* infection (TB) is an important cause of ill health, one of the top ten causes of death worldwide, and the leading cause of death from a single infectious agent (ranking above the Human Immunodeficiency Virus (HIV) [1,2]. Despite public health measures to control the infection, it still represents the leading cause of death worldwide from a single bacterial pathogen [3,4].

Healthcare workers (HCWs) present an increased risk of contracting TB through occupational exposure, such as the execution of procedures involving patients with contagious tuberculosis disease, and the manipulation of biological material contaminated with *M. tuberculosis* [5,6,7].

Following the recent epidemiological rates, latent tuberculosis infection (LTBI) affected 2.9% HCWs in low-incidence countries, up to 7.2% in high-TB-incidence countries. The introduction of TB transmission control measures may decrease TB annual incidence among HCWs [8].

According to the Italian legislation, the HCWs’ health surveillance is a fundamental tool for the prevention and control of LTBI/TB [9]. The leading healthcare interventions to preventing new infections and their progression to disease are diagnosis and treatment of LTBI [1]. Among the tests available to date for the LTBI diagnosis, tuberculin skin test (TST) and interferon gamma release assay (IGRA) represent those most frequently used [10].

Several evidences have shown that IGRAs have excellent specificity for the diagnosis of LTBI even in Bacillus Calmette–Guérin (BCG)-vaccinated populations [11,12].

IGRAs are currently being evaluated for use in the serial TB screening of HCWs [13,14,15]. Some studies have shown that the IGRAs are characterized by intra-individual variability linked to how the test is performed, reducing the reproducibility of the test, for which the use of serial tests highlights a high rate of conversions and reversions [16,17,18,19].

Different borderline zones (“grey zones”) have been proposed for defining conversions and reversions in order to improve the interpretation of test results as part of IGRA serial testing. In 2010, the Centers for Disease Control and Prevention (CDC) recommended that the role of a borderline range to improve diagnostic accuracy for QuantiFERON-TB test should be further explored [12]. Retesting of individuals with results of 0.35–0.99 IU/mL could give a solid basis for clinical interpretation and, in particular, avoid unnecessary LTBI treatment when the indication for testing is not clear [20].

This study aimed to evaluate the prevalence of LTBI among medical students and to assess the borderline zone for IGRA interpretation as a useful tool to evaluate candidate cases for chemoprophylaxis.

## 2. Materials and Methods

### 2.1. Study Design and Sample Definition

In 2011, the Occupational Medicine Departement of the University of Campania “Luigi Vanvitelli” carried out a TB mass test for HCWs management [21]. From September 2012 to November 2017, according to the health surveillance program, a sample of medical students from the University of Campania was offered TB screening.

The sample is constituted by students attending the Degree in Medicine and Surgery, the Degree Courses in Healthcare Professions, and Resident Training.

For the screening of tuberculosis infection, all medical students and postgraduates were subjected to TST (first-level exam); in case of positivity, an IGRA test, the QuantiFERON-TB^®^ Gold In-Tube assay (QFT–GIT) (Cellestis Limited, Carnegie, Australia), was performed [10].

In QFT–GIT positive cases (≥0.35 IU/mL), the investigation continued with the aim of excluding the diagnosis of active tuberculosis disease with chest X-ray (second-level exam) and specialized pneumatological consultancy (second-level exam). Subjects with a history of tuberculosis and allergy to the components of the test to be performed were excluded from the study.

Information about subjects enrolled in the sample was collected in a data card, divided into different sections, each taking into account useful data for the assessment of a possible association with an increased risk of contracting LTBI: socio-demographic characteristics, medical history such as BCG vaccination status and possible contacts with family members affected by TB, work history information, and any contact with TB patients.

### 2.2. Diagnostic Methodologies and Management of Positive Cases

The tuberculin test was performed according to the indications of the Centers for Disease Control and Prevention (CDC, 2005) by a pulmonology specialist. In accordance with the Italian guidelines [9], a wheal of at least 10 mm (or 5 mm for close contacts) was considered positive. Subjects with a positive allergic history, secondary immunodeficiency status, or pregnancy were offered the opportunity to perform the QuantiFERON-TB^®^ Gold In-Tube assay as a Level I exam.

According to the 2010 CDC guidelines, the QFT–GIT test is considered positive when interferon-gamma (IFN-γ) is ≥0.35 IU/mL after correction for the negative control. In this study, as shown in the current literature data, a borderline zone was introduced in order to better overall manage HCWs affected by LTBI, obtaining a grading as follows:

- <0.35 IU/mL: negative test;
- ≥0.35 and <1.00 IU/mL: borderline range;
- ≥1.00 IU/mL: positive test.

**Figure 1:**
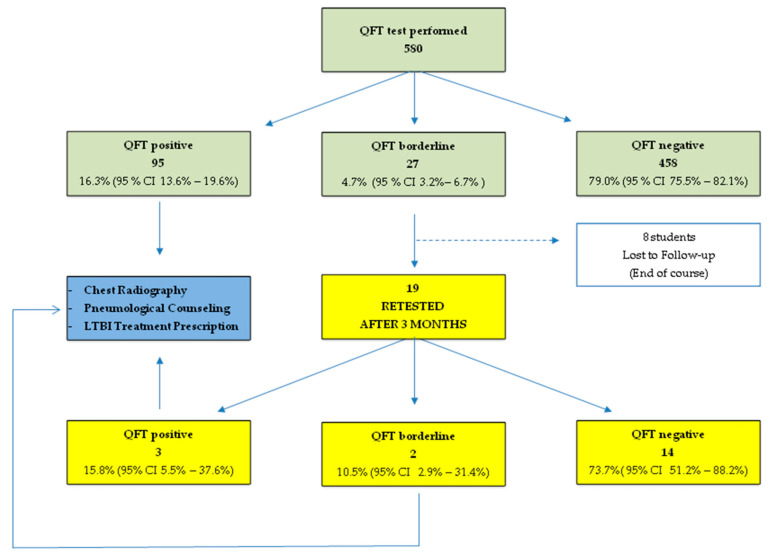
Management of the subjects that performed the QuantiFERON-TB^®^ Gold In-Tube assay (QFT–GIT). In green the first level exams; in yellow the second-level tests; in blue the third level intervention. LTBI: latent tuberculosis infection; CI: confidence interval.

A change in the (IFN-γ) concentration in the QFT result from borderline to a value of ≥1.00 IU/mL is defined as “conversion”. On the contrary, a negative change in the results to <0.35 IU/mL from a borderline value is defined as “reversion”. When simple negative/positive cutoffs are used for serial testing, issues may arise from high rates of conversions and reversions, and the higher specificity of IGRAs than of TST must be balanced against the higher probability of false-positive conversions following an initially negative test [11]. All students with first borderline IGRA results were retested within three months. Only if the second IGRA was positive were the students referred to a specialist for consultation concerning preventive chemotherapy of LTBI. The overall management scenario is represented in Figure 1.

### 2.3. Statistical Analysis

Statistical analysis of data was performed using SPSS ver. 21 (IBM SPSS Statistics, Chicago, IL, USA). Descriptive analysis and continuous variables are given as mean ± standard deviation (SD), and categorical variables as the absolute value and relative frequency. The 95% confidence intervals for proportions were calculated. After introducing the borderline zone, to evaluate the linear trend between the two test levels, a Cochran–Armitage test was performed. Statistical significance was defined as a *p*-value < 0.05.

### 2.4. Ethical Statement

The Ethics Committee of the University of Campania L. Vanvitelli approved the study protocol (ID no. 80/2018). All healthcare workers included in the survey were informed by a physician about the rationale and aims of the survey and written informed consent was obtained. Personal information regarding the subjects included in the study is protected according to Italian law [22].

## 3. Results

From September 2012 to December 2017 5468 students were enrolled in TB screening at the University Health Surveillance in Nales, Italy. Data on the cohort characteristics and medical history of the sample are reported in Table 1.

Regarding the anamnestic data, 22.6% of the students declared being an allergic subject and 12.4% suffering from at least one chronic pathology. In addition, 22 students reported having had contact with family members or patients with tuberculosis, and one of these tested positive. As for anti-tuberculosis vaccination, only 1% of the sample said they had been administered the BCG vaccine.

Foreign students enrolled in this work represent a very small percentage of the total (0.3%), and they came mainly from countries with high TB prevalence and incidence, such as Ukraine and Moldova.

Among the 5468 medical students enrolled, 5452 performed TST as a first-level exam while 16 directly performed QFT, due to some contraindications to Tuberculine test. Among 745 TST positive students, 165 did not undergo to QFT for several reasons (students coming from another university to attend a short training stage in our structures; students that left the training courses early; students that resulted in positive TST, during the screening phases at the end of training courses and which have been sent to another hospital to be managed; students affected by several diseases that could invalidate the QFT results), and 580 subjects carried out the QFT–GIT test.

Ninety-five tested positive, 458 tested negative, and 27 subjects presented within the range of values defined as borderline (0.35–0.99 IU/mL). The main characteristics of the subjects stratified by the first test results are shown in Table 2.

The QFT–GIT test was repeated after 90 days on 19 subjects with borderline values because eight students were lost to follow-up; these 19 subjects showed negativization of the values in 14 students and conversion in three cases, while for two students, a borderline value was also found for the second test. For both the conversion and borderline cases, a pneumatological counseling was deemed necessary (Figure 1).

The Cochrane–Armitage test performed showed no statistical difference between the three QFT–GIT analyzed groups (*p* = 0.67).

## 4. Discussion

Health surveillance represents a key issue for TB prevention in HCWs. For this purpose, TST and IGRA tests are currently available.

In this study, more than 5000 HCWs were initially screened by TST with a finding of about 14% of positivity and a subsequent confirmation of the IGRA test in a much lower percentage (<2%), in line with other studies conducted in European countries. In fact, this low prevalence was confirmed by other studies that found prevalence rates from 2.1% to 9.9% in German trainees [23,24] and by two studies from Denmark [25] and Norway [26] (1% and 3.4%, respectively). Our results are also in agreement with those of several Italian studies conducted among health professionals, in which the prevalence of subjects with LTBI was very low: 0.4% in a study carried out in Northern Italy by Durando et al., 1.5% in a study by Lamberti et al., and 0.62% in a study conducted by Verso et al. with both of these latter studies carried out in Southern Italy [27,28,29,30].

In recent years, several authors have focused on the fact that a simplistic dichotomy in the definition of negative and positive results of the IGRA test could be misleading due to the high numbers of spontaneous conversions and regressions [31]. Dorman et al. found that, in low endemic countries, the rate of reversion is high in subjects who tested positive for the IGRA test when the test is repeated after six months, with a significant percentage false positives among HCWs [15]. Thus, the repetition of the IGRA test could allow a correct diagnosis.

The variability over time of IGRA test results does not have a certain explanation yet. It may be due to variations in the management and reading of the test, to the variability of the immune system, and to differences in the TB activity, which can be a transient infection, infection with low replication of *M. tuberculosis* without stimulation of the immune system, or infection with high replication of *M. tuberculosis* with uncontrolled replication and stimulation of the immune system that causes an active disease [32]. Taking into account all these reasons, it is now considered appropriate to use a range of borderline values to distinguish true conversions and regressions from variations caused by the variability of the test [33].

It is, in fact, necessary to define a gray zone for QFT and to determine the range. In a study by Fong et al., 71% of conversion had IFN-γ values of ≤1 IU/mL, suggesting to extend a limit zone from 0.1 to 1.0 IU/mL [34], while Joshi et al. proposed the limit to 2.0 IU/mL since all reversions in their study had concentrations between 0.35 and 2.0 IU/mL in the first QFT [35]. In order to balance the sensitivity and specificity of QFT and to minimize conversions that could lead to unnecessary preventive treatment, Thanassi et al. proposed a limit of 1.11 IU/mL for serial QFT–GIT testing of healthcare professionals in the United States [36]. Following these recommendations, clinicians should retest low-risk individuals with an initial test result of ≥0.35 IU/mL and ≤0.7, 1.0, 1.11, or 2.0 IU/mL. Since no active TB or progression from LTBI to active TB was observed in any of the mentioned studies, it is impossible to determine which upper limit of the borderline area is to be considered.

The present study does not help to decide which limit zone is more appropriate since there is no biological reason but only a statistical one for the introduction of a limit zone for serial tests in exposed HCWs to low TB risk; from authors’ observation, it appears that the lower the boundary zone, the greater the confidence in the QFT results, according to several experts worldwide [23]. For this purpose, in this research, a borderline range of values between 0.35 IU/mL and 0.99 IU/mL has been considered. Comparing the proportions, the serial QFT-GIT results remained unchanged except for the borderliners; the application of the Cochrane–Armitage trend test resulted in no statistical significance regarding the differences found. Nevertheless, these results appeared clinically relevant due to the few positive cases. The re-evaluation of defined borderline cases, indeed, highlighted that out of a total of 19 borderline subjects retested, in only three cases was the diagnosis of LTBI confirmed. Therefore, in the present study, the interpretation of the QFT–GIT results through the use of retesting zone values had shown that in almost all students there was a change—after 90 days—with 74% regression if the positive test results fell within the range. Since active TB was not diagnosed, the application of a borderline zone extending from 0.35 to 0.99 IU/mL for QFT-GIT in this group of HCWs with a low risk of tuberculosis helped to avoid unnecessary treatment. All these findings allowed us to exclude false positives from further second-level investigations and to carry out the appropriate controls and related chemoprophylaxis only in true positive cases.

An interesting aspect would be also to consider the origin country of foreign enrolled students. In this study, the foreign enrolled students came from countries defined as high-priority countries according to the World Health Organization (WHO) “Tuberculosis surveillance and monitoring in Europe 2019”, such as Moldova (estimated number of incident cases: 95/100,000 inhabitants) and Ukraine (estimated number of incident cases: 84/100,000 inhabitants) vs. Italy (estimated number of incident cases: 7.4/100,000 inhabitants). Actually, this issue could not be well represented due to the small number of enrolled students (about 0.3%).

Although the few cohorts of students to follow up, the possible gender’s influence on the obtained results needs to be taken into account. In particular, the borderline group was composed almost entirely of women. According to the literature, the male gender is a risk factor for active TB but this gender disparity has not been fully investigated for LTBI [37].

The 27 borderline subjects were about 32 years old and were of Italian origin. Considering the current distribution of the LTBI in Italy, it mainly affects two population groups: older Italians and foreign adults (over 30 years from Eastern Europe or Northern Africa) [38]. Based on this, it appears unlikely the influence of the only gender on the outcome of the test, also remembering the small size of the enrolled sample. Further studies need to be designed with a gender-oriented analysis in order to explore this issue.

### Strength and Limitations

The study concerns a topic issue about clinical management of exposed HCW to M. Tuberculosis. Validating the borderline zone is a useful tool to avoid the third level invasive exams or treatments (e.g., X-Ray, chemoprofilaxisys). Due to the high number of screened students (>5000), this research work provides valid epidemiological support to confirm the low frequency of TB and LTBI in Italy.

However, this study presents some limitations, such as a sample size of retested subjects, and the not well gender balancing, considering that the subjects lost to follow-up were with a male prevalence. A comparison between the native and foreign population was also hampered by the sample size.

## 5. Conclusions

LTBI screening programs among HCWs are aimed at reducing the risk of transmission of *M. tuberculosis*, representing fundamental tools for TB control. Several authors have confirmed the need to introduce a limit zone for the interpretation of the IGRA in serial tests or to define a minimum increase in the concentration of IFN-γ which needs to be overcome for a conversion.

Introducing a QFT borderline range allows to select subjects who are at increased LTBI risk, identify candidates for chemoprophylaxis, and avoid unnecessary X-ray examinations and therapies. The results of this study confirm the need to define the most appropriate investigations to identify, in specific risk settings such as healthcare, the real cases of LTBI.

Further studies are needed to better address the borderline range role in discrimination between the clinical response to TB and test variability, thus improving the screening power of the QuantiFERON tests.

## Figures and Tables

**Table 1:** Demographics of the 5468 subjects enrolled.

Characteristic	Value
No. of subjects	5468
Attending:	
Healthcare profession School	2544 (46.5%)
School of Medicine	1426 (26.1%)
Postgraduate Medical School	1498 (27.4%)
Age	24.4 ± 5.3 (18–59)
Sex	
Male	2264 (41.4%)
Female	3204 (58.6%)
Nationality	
Other	16 (0.3%)
Italian	5452 (99.7%)
Vaccination against Tuberculosis	
No	5413 (99%)
Yes	55 (1%)
Family and/or Occupational Contact with Tuberculosis	
No	5446 (99.6%)
Yes	22 (0.4%)
Tubercolin Skin test	
Negative	4707 (86%)
Positive	745 (14%)
QuantiFERON^®^ Test	
Negative (<0.35 IU/mL)	458 (8.4%)
Positive (≥1.00 IU/mL)	95 (1.7%)
Borderline (≥0.35 and <1.00 IU/mL)	27 (0.5%)

**Table 2:** Characteristics of the students stratified by the first QuantiFERON-TB^®^ Gold In-Tube assay (QFT–GIT) test results.

	QFT–GIT Positive	QFT-GIT Negative	QFT–GIT Borderline
No. of students	95	458	27
Attending:			
Degree Course in Healthcare professionDegree Course in MedicinePostgraduate Medical School	33 (34.7%) 30 (31.6%) 32 (33.7%)	138 (30.1%) 122 (26.7%) 198 (43.2%)	6 (22%) 5 (19%) 16 (59%)
Age	31 ± 6.8	30 ± 6.8	32 ± 9
Sex			
Male Female	44 (46%) 51 (54%)	303 (66.2%) 155 (33.8%)	9 (33.3%) 18 (66.7%)
Nationality			
Italian Other	93 (98%) 2 (2%)	447 (97.6%) 11 (2.4%)	25 (92.6%) 2 (7.4%)
Vaccination against Tuberculosis			
No Yes	78 (82%) 17 (18%)	234 (51.1%) 224 (49.9%)	17 (63%) 10 (37%)

QFT–GIT = QuantiFERON-TB^®^ Gold In-Tube assay.

## Abbreviations

BCG	Bacillus Calmette–Guérin
CDC	lefts for Disease Control and Prevention
HCWs	Healthcare workers
IFN-γ	Interferon gamma
IGRA	Interferon gamma release assay
LTBI	Latent tuberculosis infection
QFT-GIT	QuantiFERON-TB^®^ Gold In-Tube assay
TB	*Mycobacterium tuberculosis* infection
TST	Tuberculin skin test
